# Bone Healing Improvements Using Hyaluronic Acid and Hydroxyapatite/Beta-Tricalcium Phosphate in Combination: An Animal Study

**DOI:** 10.1155/2016/8301624

**Published:** 2016-12-14

**Authors:** Yen-Lan Chang, Yi-June Lo, Sheng-Wei Feng, Yu-Chih Huang, Hsin-Yuan Tsai, Che-Tong Lin, Kan-Hsin Fan, Haw-Ming Huang

**Affiliations:** ^1^School of Dentistry, College of Oral Medicine, Taipei Medical University, Taipei, Taiwan; ^2^Dental Department, Mackey Memorial Hospital, Taipei, Taiwan; ^3^Dental Department, Wan Fang Hospital, Taipei Medical University, Taipei, Taiwan; ^4^School of Oral Hygiene, College of Oral Sciences, Taipei Medical University, Taipei, Taiwan; ^5^Dental Department, En Chu Kong Hospital, New Taipei City, Taiwan; ^6^Graduate Institute of Biomedical Materials and Tissue Engineering, College of Biomedical Engineering, Taipei Medical University, Taipei, Taiwan

## Abstract

The purpose of this study was to investigate whether the use of HLA as an aqueous binder of hydroxyapatite/beta-tricalcium phosphate (HA-*β*TCP) particles can reduce the amount of bone graft needed and increase ease of handling in clinical situations. In this study, HA/*β*TCP was loaded in commercially available crosslinking HLA to form a novel HLA/HA-*β*TCP composite. Six New Zealand White rabbits (3.0–3.6 kg) were used as test subjects. Four 6 mm defects were prepared in the parietal bone. The defects were filled with the HLA/HA-*β*TCP composite as well as HA-*β*TCP particle alone. New bone formation was analyzed by micro-CT and histomorphometry. Our results indicated that even when the HA-*β*TCP particle numbers were reduced, the regenerative effect on bone remained when the HLA existed. The bone volume density (BV/TV ratio) of HLA/HA-*β*TCP samples was 1.7 times larger than that of the control sample at week 2. The new bone increasing ratio (NBIR) of HLA/HA-*β*TCP samples was 1.78 times higher than the control group at week 2. In conclusion, HA-*β*TCP powder with HLA contributed to bone healing in rabbit calvarial bone defects. The addition of HLA to bone grafts not only promoted osteoconduction but also improved handling characteristics in clinical situations.

## 1. Introduction

Guided bone regeneration is a surgical procedure to regenerate enough bone for successful implant placement. It can be achieved by performing ridge augmentation and bone regeneration procedures which increase bone volume at bone defect areas. The materials used for guided bone regeneration should be osteoconductive or osteoinductive. Although commercialized bone graft materials have been available for some time and are proven to be a useful material for bone regeneration, there is still room for improvement.

Sinus elevation is a surgical procedure that adds bone volume to a patient's upper jaw in the area of molars and premolars. During surgery, bone graft material was packed into space where the sinus was. The most commonly used compositions for sinus elevation and guided bone regeneration is a mixture of hydroxyapatite (HA) and beta-tricalcium phosphate (*β*TCP) [1–3]. However, shaping this material to fit the sinus cavity is difficult. In addition, it is hard to adhere the material without loss during operation.

Hyaluronic acid (HLA) is a polysaccharide consisting of alternating residues of D-glucuronic acid and N-acetylglucosamine [4]. It is found in abundance in the extracellular space [5] and load-bearing joints [6, 7]. In addition, HLA is also involved in skin moisture due to its unique capacity to retain water [8]. It has been reported that HLA can be used for knee and temporomandibular osteoarthritis treatments [9]. In the field of dentistry, hyaluronic acid demonstrated anti-inflammatory, antioxidant, and antibacterial properties in the treatment of periodontal diseases [10]. In addition, due to its viscoelastic properties, it can be used as an adjunct to maintain space during the treatment of periodontitis.

Recently HLA has been studied as a biomaterial for tissue engineering. In 2011, Correia et al. prepared freeze-dried composite scaffolds of chitosan and HLA for cartilage tissue engineering and found that the incorporation of HLA enhanced cartilage ECM production [6]. In an animal study, Sasaki and Watanabe (1995) studied the osteoinductive action of HLA and found that HLA is capable of accelerating new bone formation through mesenchymal cell differentiation in bone wounds [11]. This is because bone grafts mixed with HLA can alter the physical and chemical properties of graft materials which results in enhanced capability for cell adhesion, proliferation, and migration. Their conclusion indicated that scaffolds incorporating HLA served as a support system for enhancing bone regeneration. Other recent animal studies also support the idea that composites made of bone grafts and HLA enhance bone growth and mineralization [7, 12, 13]. According to these results, Schulz et al. (2014) coated HLA on the surface of dental titanium implants and inserted them into the maxilla of miniature pigs. Their results demonstrated that HLA increases bone formation at implant/bone interface in the early healing period [4].

In 2013, ELkarargy conducted a histomorphometric study to investigate the usefulness of HA/*β*TCP with HLA for alveolar sockets preservation. He found that HA/*β*TCP with HLA exhibits a more efficient in osteoconduction when compared to the samples without HLA [14]. Bone grafts such as HA/*β*TCP used for sinus elevation and guided bone regeneration are expensive. Thus reducing the amount of the bone graft material used without affecting treatment efficiency is a challenge for scientists and dentists. Due to HLA's unique viscoelastic and osteogenetic properties, the combination of HLA and HA/*β*TCP should provide advantages compared to the use of either alone. Accordingly, the aim of this study is to test the hypothesis that an HLA and HA/*β*TCP composite material can reduce the amount of bone graft needed and increase ease of handling in clinical situations.

## 2. Materials and Methods

### 2.1. Physical Properties of HLA

The HLA used in this study is a commercially available HLA (Global Xtra, DermaFill Global, Paris, France) with a molecular weight of 2.5–3.0 MDa. The HLA was crosslinked with 1,4-butanediol diglycidyl ether (BDDE) and its concentration is 25 mg/mL. Measurement of HLA particle size distribution in this study was performed using a particle size analyzer (90 Plus, Brookhaven Instruments Corporation, Holtsville, NY, USA). Before the test, a 1 mL sample was diluted 1 : 500 with purified water and stirred for 12 hrs. The pH value of the HLA used in this study was measured using a pH-meter at room temperature (Model 6173, JENCO Quality Instruments, San Diego, USA). The rheological characteristic of the tested HLA was performed on a rheometer (RheoStress 1, Haake, Germany). The HLA properties were characterized under steady and oscillatory regimes at 25°C as a previous study [15]. Briefly, oscillatory measurements were performed at a stress of 1.88 Pa in the linear region. The frequency range and shear ratio were set at 0.1–10 Hz and 0.1–20 s^−1^, respectively. Storage (*G*′) and loss (*G*′′) moduli of the tested HLA were recorded as a function of frequency. Dynamic viscosity (*η* ^*∗*^) of the tested HLA was recorded as a function of oscillation frequency.

### 2.2. Animal Study

In this study, 6 New Zealand White rabbits, weighing 3.0–3.6 kg, were used as test subjects. The rabbits were fed solid food and water for adaptation in bracket cages at a temperature of 25°C and a humidity of 50%. All rabbits were maintained and used according to the guidelines set out in “The Care and Use of Laboratory Animals of Taipei Medical University” (LAC-2014-0087**)**. To prepare the HLA/HA-*β*TCP sample, 1 mL commercialized hyaluronic acid (Global Xtra, DermaFill, Paris, France) was diluted with 3.5 mL phosphate buffer solution (PBS). Then 100 mg HA-*β*TCP (250–500 *μ*m, MBCP, Biomatlante, Belin, France) was added to the diluted HLA solution.

**Figure 1:**
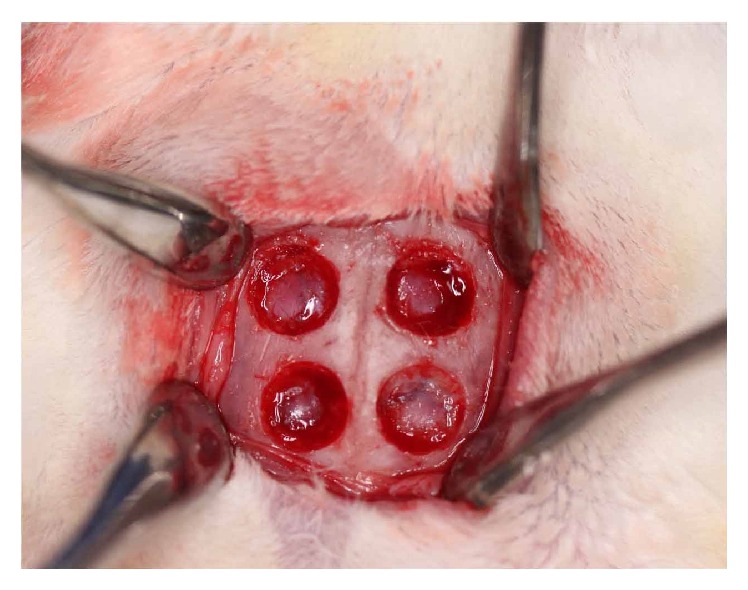
Skull defects for currently prepared material implantation. The two defects on the right were used for HLA/HA-*β*TCP and HA-*β*TCP implantation, while the two on the left were unfilled controls.

Before surgery, the rabbits were anesthetized with intramuscular Zoletil 50 at a dose of 0.5 mL (Virbac, Carros Cedex, France). The operation site was shaved and the calvaria bone was exposed through a skin incision. According to previous studies [16, 17], four circular defects with a diameter of 6 mm were prepared in the parietal bone (Figure 1). The two right defects were grafted with 0.2 g of prepared HA-*β*TCP and HLA/HA-*β*TCP. The two left defects were unfilled controls.

After 2 and 4 weeks of healing, the rabbits were euthanized under anesthesia by CO_2_ gas asphyxiation and tissues from inside the surgical sites were collected. Bone blocks were obtained using a surgical burr attached to a slow-speed electrical hand piece. The blocks were then preserved and fixed in a 10% formaldehyde solution at pH 7.0 for further analysis.

### 2.3. Micro-CT Examination

To test the new bone formation, the collected bone blocks were scanned in a micro-CT scanner (SkyScan 1076, Bruker, Kontich, Belgium). The machine was set with the following parameters: images were acquired at 49 kV, 200 *μ*A, through a 0.5 mm thick aluminum filter with a pixel size of 18.27 *μ*m. The reconstructed images were imported into the analysis software (CTAn, Bruker) for calculating bone volume. According to previous studies [18–20], the volume of interest (VOI) was defined as the relative changes in bone volume density (BV/TV%), the percentage of bone volume (BV) to the total tissue volume (TV). The new bone growth was evaluated using calculated VOI. In addition, the numbers of HA-*β*TCP particles found in the defect were counted using the micro-CT images.

### 2.4. Histological Analysis

To quantitate bone growth condition, the bone specimens were decalcified. The samples were immersed in 10% EDTA (0.1 M phosphate buffer, pH 7.4) for 4 weeks. After embedding the samples in paraffin, they were cut into 5 *μ*m thick sections. The dehydrating procedure was performed in an ascending alcohol gradient (60%–100%). Then the samples were stained with hematoxylin and eosin. Histological images were observed with a light microscope connected to a digital camera. The new bone growth condition was obtained by counting the area of newly formed bone in the defect using commercial imaging software (ImageJ, National Institutes of Health, USA). In this study, the new bone increase ratio was calculated to represent the bone growth condition. This normalized value was defined as the newly formed bone area of filled samples divided by the control sample analogue value.

### 2.5. Statistical Analysis

Mean values and standard deviations of each measurement were obtained. To evaluate differences between the sample and control, one-way analysis of variance (ANOVA) (SPSS Inc., Chicago, IL, USA) with Tukey's post hoc was performed. A *p* value lower than 0.05 was considered statistically significant.

## 3. Results

### 3.1. Physical Properties of HLA

**Figure 2:**
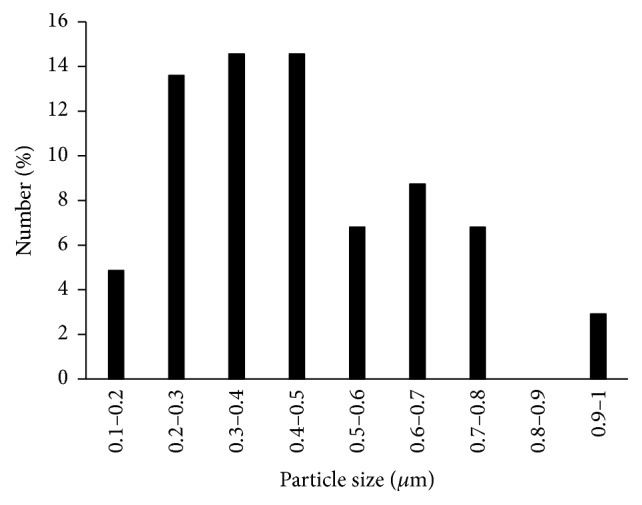
Particle diameter distribution of the HLA used in this study.

**Figure 3:**
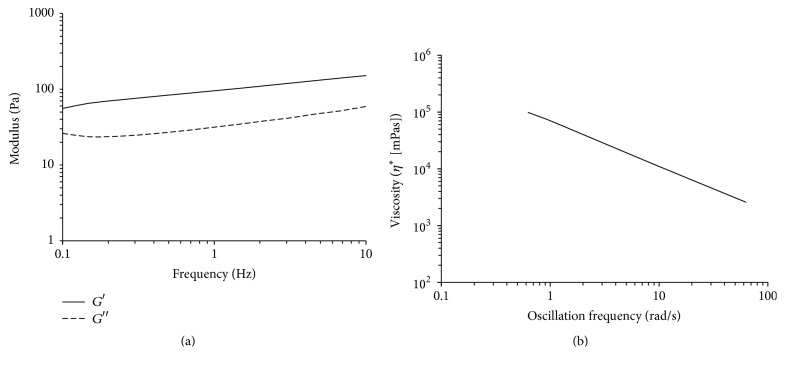
The mechanical spectra of the dynamic moduli of the HLA used in this study. Storage modulus (*G*′) is shown in graph (a) and loss modulus (*G*′′) is shown in graph (b). Graph (c) shows complex viscosity (*η* ^*∗*^) as a function of oscillation frequency.

Figure 2 shows the particle size distributions of HLA used in this study. Our results show that the diameter of HLA particles is concentrated at 0.1–0.8 *μ*m with an average of 0.36 ± 0.20 *μ*m. The pH value of HLA was 6.96 ± 0.04.  Figure 3(a) shows the mechanical tests of the dynamic moduli. The elastic modulus (*G*′) is higher than loss modulus (*G*′′). The slope of the *G*′ line is small and *G*′′ displays a frequency dependence manner.  Figure 3(b) shows the curve of complex viscosity (*η* ^*∗*^) versus oscillation frequency. The HLA demonstrates a strong shear thinning property at higher shear rates. This phenomenon indicates that the test HLA is a non-Newtonian pseudoplastic material.

### 3.2. Micro-CT Examination

**Figure 4:**
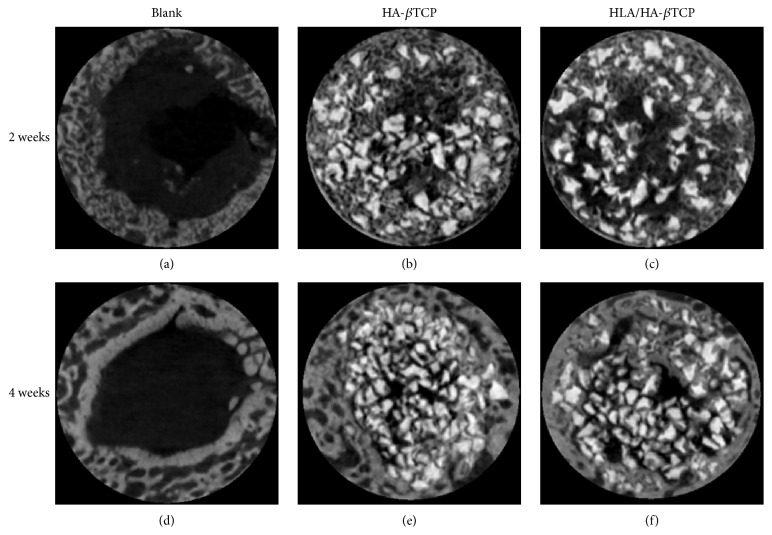
Micro-CT images of the artificial defects. (a) and (d) are the blank control at weeks 2 and 4, respectively. (b) and (e) are HA-*β*TCP filled groups at weeks 2 and 4, respectively. (c) and (f) are HLA/HA-*β*TCP filled groups at weeks 2 and 4, respectively.

Typical results of micro-CT images of bone specimens are shown in Figure 4. At both weeks 2 and 4, new bone formation can be observed in all the three groups. For the control group, newly formed bone can be found only at the area around the inner surface of the defect (Figures 4(a) and 4(d)). However, newly formed bone can be observed in the central area of the defect that was filled with HA-*β*TCP (Figures 4(b) and 4(e)). For the HLA/HA-*β*TCP samples, a similar phenomenon can also be identified (Figures 4(c) and 4(f)).

**Figure 5:**
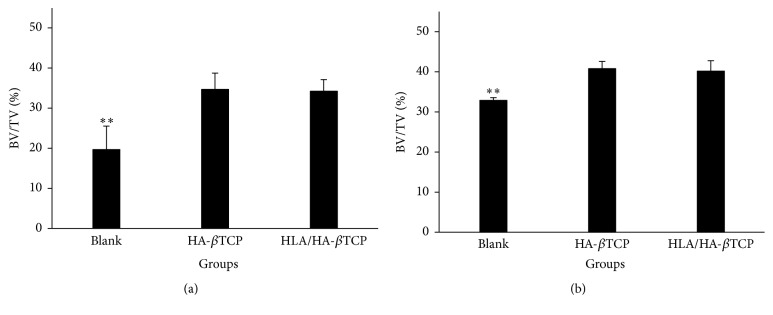
BV/TV% for the artificial defects filled with HA-*β*TCP alone and HLA/HA-*β*TCP from 2 weeks (a) and 4 weeks (b) after implantation surgery (^*∗∗*^ *p* < 0.01).

**Figure 6:**
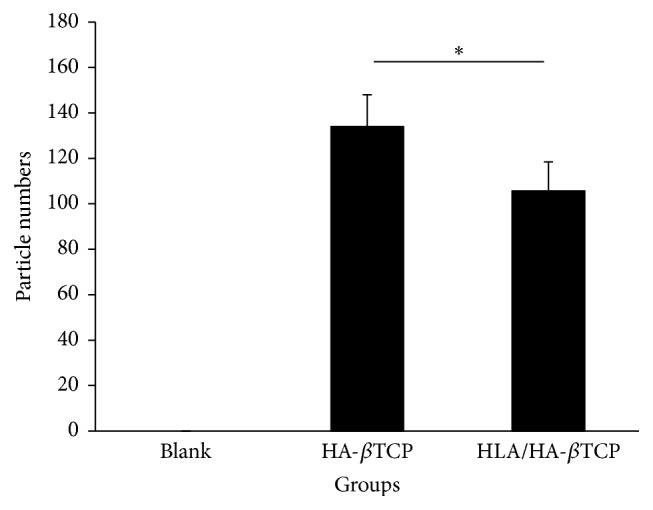
Particle numbers used in artificial defects filled with HA-*β*TCP alone and HLA/HA-*β*TCP at 2 weeks after surgery (^*∗*^ *p* < 0.05).

The differences in newly formed bone volume density (BV/TV ratio) between filled samples and controls were noted at both weeks 2 (Figure 5(a)) and 4 (Figure 5(b)) but were more obvious in the second week. However, no difference can be observed when comparing the HA-*β*TCP and HLA/HA-*β*TCP groups. At week 2, the BV/TV ratios for the HA-*β*TCP and HLA/HA-*β*TCP samples were 34.68 ± 4.04% and 34.21 ± 2.90%, respectively. These values were almost 1.7 times larger than that of the control sample (19.65 ± 5.87). At week 4, the BV/TV ratios of the HA-*β*TCP and HLA/HA-*β*TCP samples increased to 40.81 ± 1.79% and 40.18 ± 2.57%. However, there was a lower 1.25-fold difference between the filled group and blank control (Figure 5(b)).  Figure 6 shows the particles in the defect at week 2. The number of particles in the HA-*β*TCP group was 134.0 ± 14.0 which is significantly higher than that of the HLA/HA-*β*TCP group (105.7 ± 12.7). Statistical analysis revealed that this increase was significant (*p* < 0.05).

### 3.3. Histological Analysis

**Figure 7:**
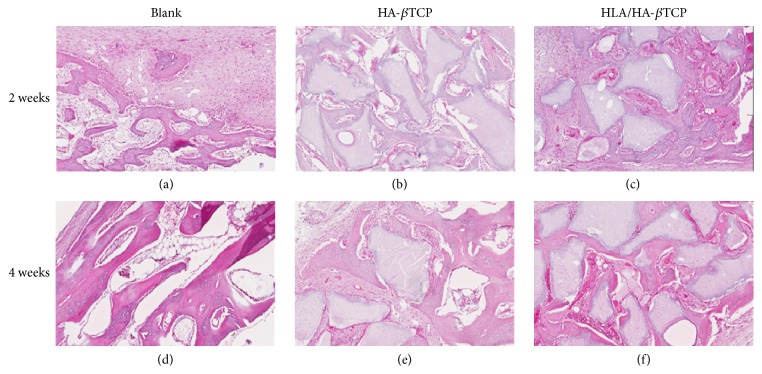
Histomorphometric images of the blank control, HA-*β*TCP alone, and HLA/HA-*β*TCP treated samples at 2 and 4 weeks.

Histological evaluation for all groups at each time point was shown in Figure 7. After 2 weeks of healing, the newly formed woven bone was in partial direct contact with the filled HA-*β*TCP surface in both the HA-*β*TCP and the HLA/HA-*β*TCP groups (Figures 7(b) and 7(c)). At 4 weeks, the newly formed bone was in close contact with the filled HA-*β*TCP surface (Figures 7(e) and 7(f)).

**Figure 8:**
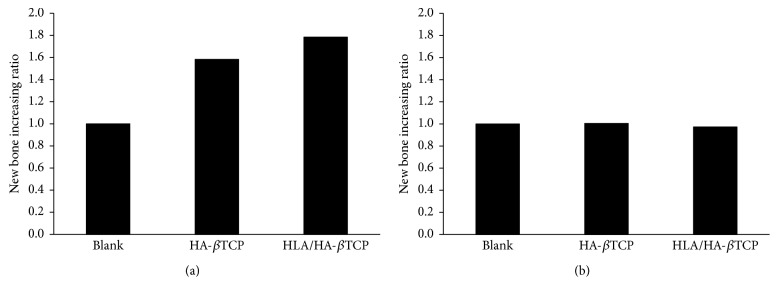
New bone increase ratio (NBIR) for the artificial defects filled with HA-*β*TCP alone and HLA/HA–*β*TCP from 2 weeks (a) and 4 weeks (b) after implantation surgery.

The histomorphometrical evaluation and the quantitative results seen in the new bone increase ratio (NBIR) are presented in Figure 8. Mean NBIR values were higher when the defect was filled with HA-*β*TCP, a 1.58-fold increase compared to the control. The ratio difference increased to 1.78-fold when HLA/HA-*β*TCP was used as the filling material. However, the NBIR values were not significantly different at 4 weeks.

## 4. Discussion

The H&E staining and micro-CT data obtained in this study showed that the prepared HLA/HA-*β*TCP had excellent biocompatibility and osteointegration (Figures 7(c) and 7(f)). Micro-CT analysis showed that the BV/TV ratio increased in both HA-*β*TCP and HLA/HA-*β*TCP samples (Figure 5). In Figure 4, significant empty space can be observed in the HLA/HA-*β*TCP filled sample. A previous report indicated that HLA in the space between bone graft powder and bone tissue may affect new bone formation [21]. However, when comparing the BV/TV ratio between HA-*β*TCP and HLA/HA-*β*TCP groups, the addition of HLA did not significantly reduce BV/TV ratio at both weeks 2 and 4 (Figure 5). The histological results showed that HLA/HA-*β*TCP-treated defects had a greater new bone increase ratio than in defects treated with HA-*β*TCP alone and untreated controls (Figure 8). This inconsistency may be due to the limitation of micro-CT, which cannot detect bone quality change until the alternation reaches 30–40% [21].

From Figures 6 and 8, we found that while the particle number for HA-*β*TCP was reduced, the bone regenerative effect remained when the HLA was present. These results provide evidence that HLA/HA-*β*TCP is useful for tissue engineering, which is consistent with previous reports suggesting that bone grafts combined with HLA enhance bone growth [7, 13, 14, 21] and mineralization [12]. From the results of micro-CT images and BV/TV ratio, a smaller number of particles were used in HLA/HA-*β*TCP group and have the same healing results compared to the HA-*β*TCP group. It is reasonable to suggest that incorporating HA-*β*TCP with HLA provides higher regenerative efficiency for bone healing.

Previous studies have also suggested that the positive effect on bone healing is found at an early stage. For example, Schulz et al. (2014) coated HLA on the surface of dental titanium implants and inserted them into the maxilla of miniature pigs [4]. They found that HLA increases bone formation at the implant-bone interface in the early healing period. An animal study by Krause et al. (2014) investigated a new bone substitute paste composed of pure phase *β*-TCP and HLA. They found that the substitute showed an early indication of bone formation [22]. In 2014, Nguyen and Lee prepared a scaffold by loading HLA hydrogel into a biphasic calcium phosphate (BCP) ceramic. After a series of animal studies, they suggested that this novel bone substitute exhibited rapid new bone formation and a high rate of collagen mineralization [12]. In the present study, the BV/TV ratio for the HLA/HA-*β*TCP filled samples was almost 1.7 times larger than that of the control sample at week 2. However, the difference between ratios was 1.25 at week 4. This phenomenon confirmed the results of previous studies which indicated that the positive effect of HLA on bone healing is seen at an early healing period because HLA is one of the components of the extracellular matrix that serves as a scaffold for mesenchymal cell migration [23]. This effect induced the mesenchymal cells to differentiate, proliferate [11, 24], and migrate [25] which induced growth of osteoblasts and osteocytes at the early healing stage.

It has been reported that HLA's regenerative function is strongly affected by its physical properties. The HLA used in this study is a commercially available crosslinked product with high molecular weight. According to a previous study, the reticulated HLA can demonstrate a better regenerative function compared to linear HLA when it is mixed with *β*TCP granules [26]. In addition, particle size and molecular weight also strongly affects the biofunction of HLA [8, 27]. In 2015, Zhao et al. found that high molecular weight HLA increased the mRNA expressions of ALP, RUNX-2, and OCN [28]. That is, HLA of higher molecular weight promoted bone formation. It is well known that high molecular weight and crosslinking degree result in high HLA viscosity.  Figure 3(a) shows mechanical tests of the dynamic moduli. The elastic *G*′ is higher than *G*′′. In addition, the slope of the *G*′ line is small and *G*′′ displays a minimum at intermediate frequencies dependence manner as previously reported [14]. This result demonstrates that the HLA used in this study is a gel with high viscosity. This phenomenon suggests that the HLA used in this study may provide bone growth effect as mentioned above. Although HA and *β*TCP granules are commonly used biomaterials for repairing bone defects, when they are dried these granules are difficult to handle in the surgical room because of low weight and lacking cohesion [26]. Incorporating these bone graft particles into a hydrogel could be a possible way to solve this problem. From the result, we concluded that incorporating HA-*β*TCP with HLA could be a satisfactory method for improving both regenerative efficiency and ease of handling [29–31].

## 5. Conclusion

In conclusion, the HLA/HA-*β*TCP present in this study provides bone regeneration in situations with a low amount of HA-*β*TCP granules. In addition, this novel material provides handling efficiency during the surgical process. Overall, HLA/HA-*β*TCP exhibits great promise for use in stimulating new bone formation for the treatment of sinus elevation and guided bone regeneration.
